# Multiple-level stakeholders’ perspectives on barriers and facilitators of HPV vaccination delivery in Western and Central Kenya: a qualitative study

**DOI:** 10.1136/bmjph-2026-005069

**Published:** 2026-10-08

**Authors:** Emmah Owidi, Lynda M Oluoch, Bryan J Weiner, Cynthia Cheptoo, Christine W Miano, Lucy Mecca, Lucy Kiarie, Ezinah Nyangena, Belitah Nzuki, Paul Mwangi, Betty Njoroge, Imeldah Wakhungu, Maricianah Onono, Elizabeth A Bukusi, Rose Jalang’o, Kenneth Ngure, Ruanne V Barnabas, Nelly Mugo, Grace Umutesi

**Affiliations:** 1 Centre for Clinical Research - Partners in Health and Research Development, Kenya Medical Research Institute, Nairobi, Kenya; 2 Department of Global Health, University of Washington, Seattle, Washington, USA; 3 National Vaccines and Immunization Program, Kenya Ministry of Health, Nairobi, Kenya; 4 Johns Hopkins Program for International Education in Gynecology and Obstetrics (JHPIEGO), Nairobi, Kenya; 5 Center for Clinical Research, Kenya Medical Research Institute, Nairobi, Kenya; 6 Center for Microbiology Research, Kenya Medical Research Institute, Nairobi, Kenya; 7 Department of Obstetrics and Gynecology, University of Washington, Seattle, Washington, USA; 8 School of Public Health, Jomo Kenyatta University of Agriculture and Technology, Nairobi, Kenya; 9 Division of Infectious Diseases, Massachusetts General Hospital, Boston, Massachusetts, USA; 10 Harvard Medical School, Boston, Massachusetts, USA

**Keywords:** Human Papillomavirus Viruses, Public Health, Primary Prevention, Qualitative Research, Vaccination

## Abstract

**Background:**

Human papillomavirus (HPV) vaccination is critical for achieving cervical cancer elimination. In Kenya, HPV vaccination coverage progressed slowly since the launch of the national vaccination programme in 2019. We sought to assess the barriers and facilitators of HPV vaccination delivery from the perspective of multilevel stakeholders involved in HPV vaccination in Kenya.

**Methods:**

Using a qualitative approach, we interviewed multilevel stakeholders involved in vaccination delivery across multiple levels of the health system in three counties and nationally between February and July 2023. Participants were purposively sampled based on their role in HPV vaccination delivery. We used in-depth interview guides informed by the consolidated framework for implementation research. Interviews were audio-recorded, transcribed and translated into English when necessary. Data were analysed using deductive and inductive thematic approaches.

**Results:**

Overall, we interviewed 52 participants, including 22 (42%) community leaders, 19 (37%) healthcare providers and 11 (21%) decision-makers. 34 participants (65%) were females. Participants had been in their role for 9 years on average (IQR: 3–12). Stakeholders reported that strong global, regional and local evidence supported national implementation of HPV vaccination. Adaptations to the intervention, including school-based and community-based vaccination, complemented facility-based approaches and increased coverage. Additionally, the availability of vaccination resources, together with adequate stakeholder engagement, training and motivation, was critical for HPV vaccination delivery. Conversely, training gaps and limited dissemination of evidence to key stakeholders hindered vaccination delivery. Furthermore, logistical constraints, poor coordination and communication among stakeholders and the lack of parental consent challenged school-based delivery. Facility-based vaccination was impeded by inadequate infrastructure and staffing, while community hesitancy hindered vaccination uptake due to inadequate knowledge, insufficient early involvement of key community stakeholders, myths and misinformation.

**Conclusions:**

Context-specific approaches that include cross-sectoral collaboration efforts among HPV vaccination stakeholders and targeted sensitisation within communities could improve HPV vaccination delivery and uptake in Kenya and comparable settings.

**WHAT IS ALREADY KNOWN ON THIS TOPIC**

- Global studies have shown that human papillomavirus (HPV) vaccination is a safe and effective strategy to prevent cervical cancer by preventing cancer-causing HPV infections. Despite the high burden of cervical cancer in Kenya, HPV vaccination coverage remains low, highlighting the need for evidence to inform delivery strategies that will improve coverage and protect as many lives as possible.

**WHAT THIS STUDY ADDS**

- This study is the first to systematically apply the consolidated framework for implementation research to identify contextual factors influencing HPV vaccination delivery in Kenya. By drawing on the perspectives of different stakeholders across multiple levels of the healthcare system, this study offers actionable strategies to address barriers and leverage facilitators to improve HPV vaccination uptake in Kenya.

**HOW THIS STUDY MIGHT AFFECT RESEARCH, PRACTICE OR POLICY**

- The findings of this study provide timely evidence that could inform the design and implementation of Kenya’s national HPV vaccination strategies and guidelines.

## Introduction

 Low- and middle-income countries (LMICs) accounted for up to 90% of cervical cancer cases reported globally in 2022.^1,2^ In 2020, 21% of all cervical cancer-related deaths occurred in sub-Saharan Africa.^3,4^ These high proportions are attributed to the high disease burden, low awareness of prevention services, inadequate access to quality healthcare and delayed diagnosis and treatment.^5–7^ Human papillomavirus (HPV) is the primary cause of cervical cancer, but HPV vaccination is a highly effective preventive strategy when administered before sexual debut.^4,8–10^ This prompted the WHO to recommend that countries vaccinate at least 90% of girls before the age of 15 years.^11,12^

Since 2006, many countries have launched national HPV vaccination programmes targeting girls aged 9–14 years, using two-dose or three-dose HPV vaccination schedules at 6-month intervals.^13–15^ Common strategies for delivering HPV vaccination have included school-based vaccination, facility-based vaccination, community-based vaccination or integrated approaches that combine different strategies.^16–18^ In Kenya, cervical cancer was the second most common cancer among women and the leading cause of cancer-related deaths among women in 2022.^3^ Kenya adopted an integrated delivery approach that includes facility-based, school-based and community-based vaccination for girls aged 10–14 years to reach both school-going and out-of-school girls.^4,19^ However, numerous barriers still hinder HPV vaccination delivery and uptake in Kenya, where vaccination coverage was only 33% in 2023.^2,20,21^ Key barriers include low knowledge and misconceptions about HPV vaccination, limited accessibility to healthcare facilities and financial constraints.^22–25^

Despite recent progress, gaps remain in understanding the drivers of HPV vaccination from different stakeholders’ perspectives. We sought to understand contextual factors that influence HPV vaccination delivery from the perspectives of multilevel stakeholders involved in vaccination delivery at the national and county levels in Kenya to inform future strategies for improving HPV vaccination coverage and broader vaccination efforts.

## Methods

### Study design and setting

This qualitative study was nested within the KENya Single-dose HPV-vaccine Efficacy study (KEN SHE: ClinicalTrials.gov NCT03675256). The KEN SHE study is a randomised, multicentre, double-blind, three-arm, controlled trial that sought to test the efficacy of single dose bivalent and nonavalent HPV vaccines.^26^ We sought to generate insights on drivers of HPV vaccination in Kenya.

### Study population and sampling

Participants were recruited from the national level and in Kiambu, Nairobi and Kisumu counties where the KEN SHE study was conducted.^27^ Participants were purposively sampled based on their role in HPV vaccination delivery to capture perspectives across multiple levels of the healthcare system. Participants included local administration leaders, religious leaders, community health promoters (CHPs), teachers, healthcare providers (HCPs), programme implementers and policymakers. Eligible participants were invited to participate via email or phone calls. Others were recruited through referral by previously contacted stakeholders, and interviews were conducted until data saturation was reached.^28^

### Data collection

Interviews were conducted from February to July 2023 and lasted approximately 1 hour. The interviews were conducted in-person or remotely via video conferencing (Zoom), by four experienced female qualitative researchers in the participants’ preferred language (English, Swahili or local dialects). Remote interviews yielded high-quality data despite occasional internet connectivity challenges. We used a semistructured interview guide developed using the consolidated framework for implementation research (CFIR) for LMICs (online supplemental file 1).^29^ CFIR has five broad domains: intervention/innovation, outer setting, inner setting, individuals and implementation process. The CFIR for LMICs includes an additional systems domain (figure 1). We selected CFIR due to its wide application as a theoretical framework for assessing barriers and facilitators to the success of implementation interventions.^30^ Additional details on the study design and data collection have been described elsewhere.^27^

**Figure 1:**
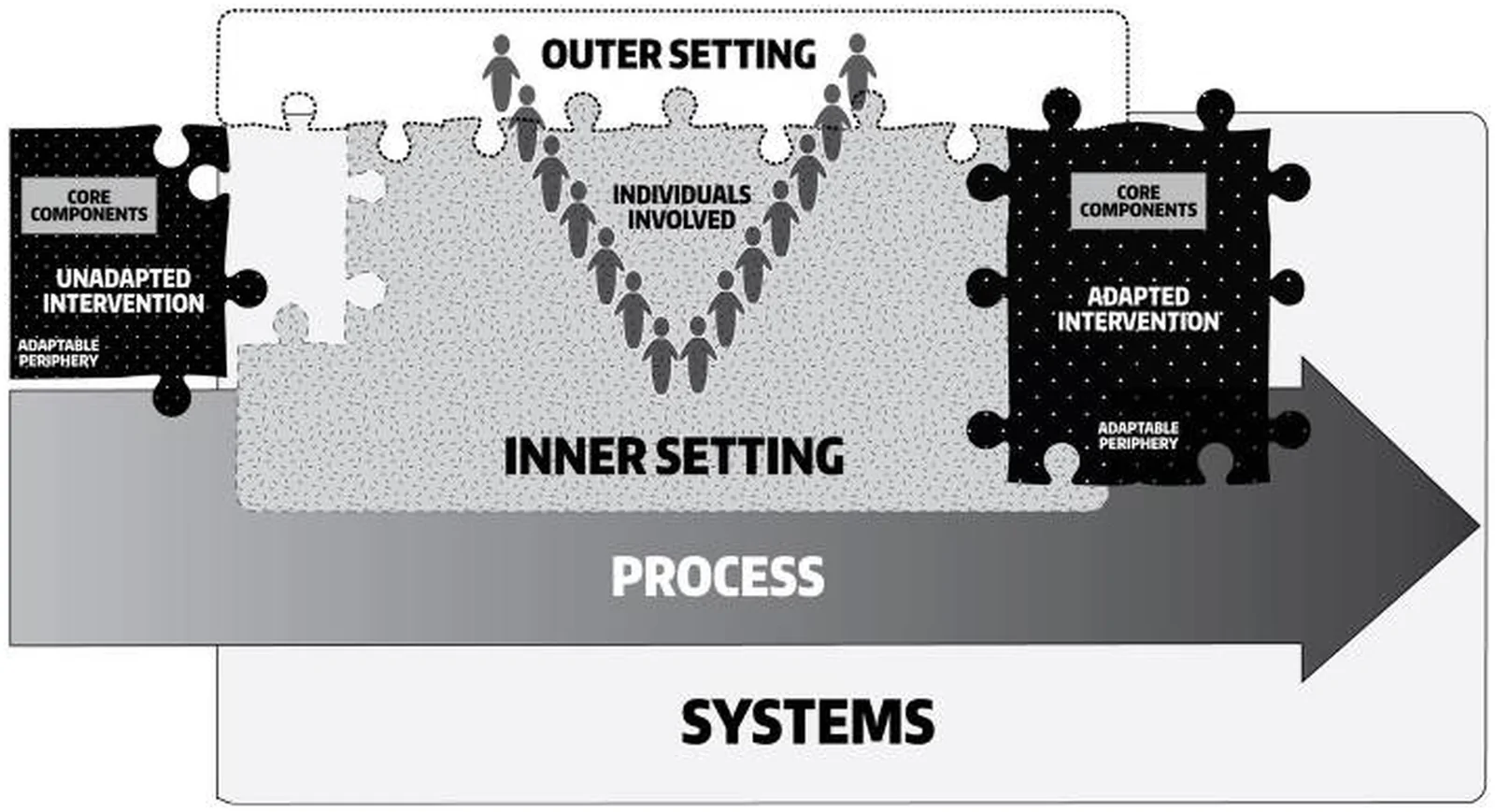
The CFIR framework for LMICs. CFIR, consolidated framework for implementation research; LMIC, low- and middle-income countries.

### Data management and analysis

All interviews were audio-recorded, transcribed and translated into English where necessary. After each interview, interviewers summarised key points in debriefing reports. Interviews were analysed using deductive and inductive thematic approaches.^31^ Initial coding was guided by CFIR (deductive), with emergent insights iteratively mapped onto relevant CFIR constructs (inductive). All analyses were performed on de-identified data using Dedoose V.10.0.59 (Sociocultural Research Consultants, Los Angeles, California, 2025).^32^ Two team members (EO and CC) reviewed all transcripts and developed a codebook based on the updated CFIR framework and the interview guides (online supplemental file 2).^33^ Four team members (EO, CC, EN and BN) selected a subset (15%; n=3) from each category and double-coded them to ensure consistency. Codes were reviewed and discrepancies resolved during regular meetings until agreement was reached. The remaining transcripts were coded independently. Key themes from the interviews were summarised and findings organised across CFIR constructs.

The Standards for Reporting Qualitative Research (SRQR) and the SRQR reporting checklist were used to draft and edit the manuscript (online supplemental file 3).^34,35^

### Ethical statement

This study was approved by the institutional review boards of the Kenya Medical Research Institute (Protocol No. KEMRI/SERU/CCR/0283/4546), the University of Washington (IRB ID: STUDY00016065) and Massachusetts General Hospital (Protocol No. 2022P002863). Informed consent was obtained from all participants.

### Patient and public involvement

This study was conducted in collaboration with the National Vaccines and Immunization Program (NVIP) and involved stakeholders across different levels of the Kenyan healthcare system. These stakeholders provided insights that informed study implementation, including participant selection, referral and recruitment.

## Results

### Participant characteristics

Overall, 52 participants were interviewed. Of these, 34 (65%) were female, and on average, participants had been in their roles for 9 years (IQR: 3–12). Participants were involved in HPV vaccination delivery at the national level (n=4), Kiambu (n=15), Kisumu (n=19) and Nairobi county (n=14). Additionally, 22 (42%) were community leaders, 19 (37%) were HCPs and 11 (21%) were county-level and national-level decision-makers (table 1).

**Table 1:** Participant demographics characteristics (n=52)

	Kiambu (n=15)	Kisumu (n=19)	Nairobi (n=14)	National (n=4)	Overall (n=52)
Stakeholder group					
Community leaders*	7 (46.7%)	8 (42.1%)	7 (50.0%)	0 (0.0%)	22 (42.3%)
Healthcare providers†	5 (33.3%)	8 (42.1%)	6 (42.9%)	0 (0.0%)	19 (36.5%)
Decision-makers‡	3 (20.0%)	3 (15.8%)	1 (7.1%)	4 (100.0%)	11 (21.2%)
Sex					
Female	10 (66.7%)	12 (63.2%)	9 (64.3%)	3 (75.0%)	34 (65.4%)
Male	5 (33.3%)	7 (36.8%)	5 (35.7%)	1 (25.0%)	18 (34.6%)
Duration in role					
Mean (SD)	12.7 (12.0)	9.5 (6.4)	7.0 (5.7)	8.3 (4.6)	9.7 (8.3)
Median (Q1, Q3)	10.0 (5.5, 13.5)	10.0 (3.5, 12.5)	5.0 (3.5, 9.5)	8.5 (5.3, 11.5)	9.0 (3.0, 12.3)

* Included seven religious leaders, three local administration leaders, seven community health promoters and five teachers.

† Included 15 nursing officers, three nursing officers in charge and one principal nursing officer.

‡ Included seven Ministry of Health officials, 2 Ministry of Education officials and two staff from Johns Hopkins Program for International Education in Gynecology and Obstetrics and John Snow, Inc.

Q1, first quartile; Q3, third quartile.

Participants highlighted multiple factors across CFIR domains that influenced HPV vaccination delivery in Kenya, as described in the subsequent sections and summarised in table 2.

**Table 2:** Summary of key insights from stakeholders, organised by CFIR domains and constructs

Domain	Construct	Barriers	Facilitators	Recommendations
Innovation	Evidence-base	Uneven evidence dissemination; uncertainty about effectiveness among some stakeholders	Strong policy and clinical rationale; Kitui pilot success cited as proof of concept	Develop easy-to-use evidence briefs; integrate survivor stories/local data
	Design	Insufficient, non-localised IEC; late/uneven distribution; limited visual aids	SOPs/registers exist; some posters available in facilities	Co-create culturally tailored IEC; disseminate early
	Adaptability	Rigid consent processes; difficult access in private/gated schools; limited youth-friendly options	Multiplatform delivery (eg, schools, facilities, churches/mosques)	Offer weekend/after-school sessions; tailor strategies by school type/context; integrate with adolescent services
	Complexity	Two-dose schedule, consent logistics cause drop-offs and missed opportunities	School-based delivery effective when well organised	Strengthen defaulter tracking; align to school calendar; simplify standardised consent
	Cost	Outreach resource needs (eg, transport, staff allowances); private facilities charge fees (equity issue)	Free vaccination in public sector; partner support offsets some costs	Budget for predictable outreach costs and small stipends; negotiate reduced/standardised private sector pricing
Outer setting	Critical incidents	COVID-19 disruptions; competing priorities; vaccine hesitancy linked to COVID-19 misconceptions	Catch-up strategy introduced for older girls	Establish clear communication strategies and messaging; normalise HPV vaccination as routine
	Local attitudes	Parental consent refusals; myths; girls’ fear of injections; limited sensitisation; privacy concerns; time/transport constraints	Positive peer influence among girls; schools/churches as outreach platforms	Provide parent-first briefings; private, age-appropriate spaces; peer counsellors; address adverse event queries upfront
	Policies and laws	School-access bureaucracy; mixed/late directives on consent	Existing WHO guidelines; inclusion of HPV vaccination in national immunisation and school health policies	Harmonise national consent guidance; embed HPV vaccination in school health programmes
Inner setting	Structural characteristics	Few adolescent-friendly rooms; space constraints; staffing shortages; cold-chain gaps; hard-to-reach schools/roads	Existing EPI infrastructure; availability of facility/school/community spaces	Designate adolescent-friendly spaces; plan mobile/outreach with transport pools; ensure cold-chain coverage at outreach hubs
	Relational connections and communications	Top-down information flow; late engagement of teachers/CHPs; misinformation persists	Stakeholder meetings, TWGs, WhatsApp groups enable rapid coordination	Include teachers/CHPs in microplanning and term calendars; provide simple talking-point kits
	Compatibility and relative priority	Lower priority versus COVID-19/HIV/malaria; limited incentives for staff; weak goals/targets and feedback locally	Integration in facilities feasible; basic tools available; reviews/recognition exist	Set clear monthly targets per school/facility; fund outreaches; provide micro-incentives (transport/lunch) for outreaches
	Available resources	Gaps in transport, staffing, IEC and budgets	Vaccines and basic tools usually available	Fund outreach activities in AWPs
	Access to knowledge and information	Knowledge gaps exist among key stakeholders (eg, teachers, CHPs, HCPs, parents); myths/misconceptions exist	Initial training provided; refresher training needed	Schedule periodic CME/refreshers for HCPs, teachers, CHPs
Individuals	Capability	Some stakeholders feel underprepared; knowledge gaps among HCPs/teachers/parents	HCPs/CHPs conduct advocacy; supportive parents/teachers; CHPs/teachers often confident mobilisers when supported	Provide concise fact sheets and FAQs; train opinion leaders; equip teachers with class talk scripts; offer brief, skill-based micro-trainings (myth-busting, parent dialogues)
	Motivation	Early scepticism among some leaders; limited staff motivation without allowances/logistics; limited formal recognition for champions	Progression from scepticism to advocacy after training/evidence dissemination; survivors, teachers, CHPs act as champions	Identify and formally recognise champions; provide predictable outreach support; provide small resources (eg, airtime, print kits) to sustain activity
Implementation process	Planning	Rushed initial rollout; short-notice outreach; limited written plans; uneven involvement of gatekeepers	Mapping schools to facilities; county/subcounty coordination structures exist	Co-develop annual micro plans aligned to school terms; schedule parent consent drives ahead of outreaches; pre-position vaccination supplies
	Engaging	Late/limited engagement of teachers/CHPs/community and religious leaders; variable partner alignment	Multisectoral forums (MOH-MOE-Ministry of Interior-NGOs); community dialogues; use of peer educators	Run termly joint forums with headteachers/chiefs; assign explicit roles to stakeholders (eg, referral, mobilisation, defaulter tracing)
	Doing	Staff/transport gaps; consent bottlenecks; weak second-dose follow-up	Mixed model (facility+school +community) works when adequately resourced; WhatsApp groups facilitate stakeholder coordination	Create standard outreach kits (eg, transport, allowances, IEC, defaulter lists); book adequate time slots per school; integrate with other youth services
	Reflecting and evaluating	Data move upwards but limited local feedback/targets; inconsistent review forums	KHIS/DHIS2 reporting; quarterly reviews influence additional outreach planning	Establish monthly school linked dashboards for facilities; set/track explicit coverage targets; after-action reviews/close feedback loop with schools/CHPs
Characteristics of systems	Systems architecture	Devolved service delivery funding gaps; limited county budgets; donor dependency	Partner/donor co-financing; political goodwill	Allocate dedicated county HPV vaccination budgets
	Strategic policy alignment	Limited knowledge among community leaders; insufficient data on eligible girls hinders planning	Strong political and leadership support; proactive counties facilitate implementation	Institutionalise MOH-MOE collaboration; quarterly intercounty learning exchanges; conduct joint microplanning at subcounty level

AWP, annual work plan; CHP, community health promoter; DHIS2, District Health Information System 2; EPI, Expanded Programme on Immunization; FAQ, frequently asked questions; HCP, healthcare provider; HPV, human papillomavirus; IEC, information, education and communication; KHIS, Kenya Health Information System; MOE, Ministry of Education; MOH, Ministry of Health; NGO, non-governmental organization; SOP, standard operating procedure; TWG, technical working group.

### Innovation

#### Innovation evidence-base

Stakeholders reported that Kenya’s HPV vaccination introduction was informed by strong global, regional and local evidence from vaccine efficacy and safety studies. Evidence on national cervical cancer statistics, economic, health and social impacts and positive outcomes from early-adopter countries were shared with policymakers and HCPs. This evidence reinforced stakeholders’ confidence in the potential of HPV vaccination to reduce the burden of cervical cancer, mortality and treatment costs.

We have been having so many cases of cervical cancer and poor outcomes have come because of late diagnosis and lack of adequate treatment facilities at every level. So, having a vaccine that would prevent women from getting cervical cancer…it becomes something of necessity and also timely. (Principal nursing officer, Kisumu county).

The WHO endorsement and familiarity with local studies such as KEN SHE strengthened trust in HPV vaccination among HCPs, community leaders, teachers and health promotion officers. Furthermore, HCPs believed that HPV vaccination was safe because they did not observe adverse reactions during early implementation. Additionally, the Kitui pilot project confirmed the feasibility and acceptability of school-based HPV vaccination, which guided vaccination rollout. However, limited dissemination of detailed evidence on vaccine safety and effectiveness affected confidence among HCPs and CHPs.

#### Innovation design

HPV vaccination was implemented routinely in healthcare facilities and integrated into the national Expanded Programme on Immunization (EPI). The vaccination was provided alongside other childhood vaccines in child welfare clinics within maternal and child health (MCH) clinics.

In all health facilities, we have welfare clinics for our children. Right from birth, those are the preferred points of vaccination. So, when we implemented the HPV vaccine into the routine [services]…this is…where they would also go and get the vaccination. (National-level decision-maker).

However, stakeholders reported limited uptake of facility-based HPV vaccination because eligible girls rarely visited facilities. Additionally, most MCH clinics operated only during standard working hours, limiting access during evenings and weekends. Limited demand-creation further affected facility-based vaccination, occasionally resulting in expired vaccines.

#### Innovation adaptability

School-based and community-based HPV vaccination complemented facility-based vaccination. School-based vaccination was promoted through engagement with schools and teachers. Consent was obtained by asking girls to deliver letters to their parents and return signed letters to their schools. In some counties, schools and institutions (eg, churches) were linked to nearby facilities for community mobilisation and outreaches. Stakeholders perceived school-based vaccination as more effective than facility-based vaccination because it reached larger numbers of age-eligible girls (10–14 years).

We can do outreaches in schools because most of the population that we target is found in school. The hours we want to vaccinate them…they’re in school during those hours…We also have a population that is out of school…We also target them in terms of outreach in the community. (National-level decision-maker).

Community outreaches and rapid results initiatives, including those integrated into health campaigns conducted in markets, churches and community halls, were also effective in reaching girls, including out-of-school girls. However, staffing, logistical and consenting challenges and delays or failure in revisiting schools for second doses hindered delivery.

#### Innovation complexity

Participants perceived facility-based HPV vaccination and documentation to be simple and quick, similar to other childhood vaccinations. However, communities’ lack of knowledge, misconceptions about HPV vaccination and difficulties in reaching target populations complicated delivery. Notably, parents rarely took age-eligible girls to facilities for vaccination because they were not the usual target group for routine immunisation.

The only challenge is the clientele base…in a month, you will get maybe like two or three clients who have just walked in…And having also other services…maybe one nurse needs to administer other services. (Decision-maker, Kiambu county).

School-based vaccination was considered more complex than facility-based delivery because of the involvement of multiple sectors and stakeholders, including the Ministry of Health (MOH), Ministry of Education (MOE), non-governmental organisations (NGOs), CHPs and religious and political leaders. The multistep implementation process, which involved community mobilisation, parental consenting, and follow-up for second doses, further complicated logistics. Additionally, outreaches were resource-intensive, requiring coordination with schools, transportation, allowances and additional staff.

#### Innovation cost

While public healthcare facilities provided HPV vaccination at no cost, private facilities charged a fee (eg, KES 600 (~US$5) per dose). During outreaches, HCPs incurred expenses (eg, transportation, lunch and refreshments) which were sometimes reimbursed. School-based vaccination was perceived to be costly because of the resources required for outreaches, stakeholder engagement, sensitisation, continuous advocacy and community mobilisation.

There are some schools that we have gone, and we have given the first dose of the HPV vaccine but once the six months are over, we are not able to go back there, and not that we don’t have vaccines, the reason is, we don’t have reimbursement for lunch. (Nursing Officer, Nairobi county).

Limited funding restricted training among all relevant stakeholders, and some counties prioritised which stakeholders would receive training. Policymakers perceived facility-based vaccination as more sustainable than school-based strategies, which influenced the adoption of facility-based vaccination as the primary national delivery strategy.

### Outer setting

#### Critical incidents (COVID-19)

HPV vaccination was integrated into COVID-19 vaccination outreaches to leverage available staffing and resources. Additionally, the COVID-19 pandemic influenced the introduction of an HPV vaccination catch-up strategy targeting girls aged 10–14 years, instead of the original strategy that targeted only girls aged 10 years. This approach sought to reach eligible girls who had been missed because of the COVID-19-related social restrictions in 2020–2021.

However, COVID-19 restrictions disrupted school-based HPV vaccination because of school closures. COVID-19 vaccine hesitancy further reduced HPV vaccination uptake because of spillover effects from hesitancy towards COVID-19 vaccination. Additionally, competing priorities from the COVID-19 response diverted resources and attention from HPV vaccination.

COVID vaccination…was given a high priority…it kind of stopped all the services because…we used to close other services like family planning which is not an emergency…So, you could find that even a nurse working in the family planning has gone to do COVID vaccination. (Nursing officer, Nairobi county).

#### Local attitudes

Stakeholders reported overall good acceptance of HPV vaccination, especially among communities that received adequate information. Stakeholder support for HPV vaccination was linked to adequate involvement, training and sensitisation. Acceptance at the community level was based on trust built through adequate engagement with local leaders. Community leaders and gatekeepers (eg, administration leaders, religious leaders and teachers) were trusted and influenced community acceptance of HPV vaccination.

However, community hesitancy was largely driven by limited awareness and persistent myths and misconceptions about the HPV vaccine (eg, that it is a contraceptive, causes infertility or is being used for trials in girls). A lack of trust in government programmes and limited knowledge among girls and parents also fueled hesitancy, resulting in some parents refusing to provide consent for their daughters’ HPV vaccination.

Those with the signed consent are the ones who were vaccinated. We really vaccinate many girls when we go to public schools but if we go to private schools, we find other parents refuse to sign the consent. (Community health promoter, Kiambu county).

Resistance from religious and cultural leaders who claimed that HPV vaccination was laced with contraceptives to reduce the population further influenced community hesitancy. Hesitancy was also linked to concerns about vaccine safety and quality (eg, that it was unsafe because it was free or expired and possible side-effects or counteractions with other medications) and concerns about vaccinating only girls. Among girls, fear of injection pain, lack of parental consent, social media misinformation and messaging that HPV vaccination was administered only to girls before sexual debut created stigma and fueled vaccine refusal.

#### Policies and laws

International and national policies and guidelines, including the WHO Cervical Cancer Elimination Strategy and Kenya’s HPV vaccination and school health policies, supported implementation. Initial WHO guidance informed the integration of HPV vaccination into routine immunisation in response to Kenya’s high burden of cervical cancer. Leveraging existing governance structures further enhanced implementation effectiveness.

In government it becomes difficult to begin new structures, so it’s good to leverage on the existing structures…if they are working, why not work with them than taking forever to start new structures which may not work? (National-level decision-maker).

However, bureaucratic hurdles (eg, need for MOE clearance to access schools), lack of consistent NGO support and parental consent requirements impeded HPV vaccination. Unlike other school-based health campaigns that did not require parental consent, teachers feared prosecution if parents reported them for allowing their children to be vaccinated without parental consent.

They [teachers] also fear, like for Nairobi, they get reported…You have…allowed my child to be vaccinated without my consent. But for routine [vaccination], I have never heard somebody complain that we visited a school for the polio campaign. (Decision-maker, Nairobi county).

### Inner setting

#### Structural characteristics

Existing space within facilities and EPI infrastructure (eg, cold-chain, refrigerators, data tools) facilitated HPV vaccination delivery and integration. However, inadequate space to accommodate large numbers of girls hindered delivery. Shortages of trained staff, heavy clinic workloads and limited ownership of vaccination roles further hindered delivery, especially when key staff were unavailable. Additionally, staff shortages made it difficult to balance routine responsibilities with outreaches, disrupting services, particularly in private facilities.

For the private [facilities], sometimes it is difficult. Like now…here I am alone. So, it is hard for me to go for an outreach in the schools but for the public, maybe if they [staff] are usually many, one of them can go and leave the other one. (Nursing officer, Kiambu county).

The lack of cold-chain equipment and refrigerators, especially in lower-level facilities, further limited access to vaccination. The use of manual registers due to a lack of digital tracking systems increased provider burden, while poor road networks and difficult terrain hindered outreaches.

#### Relational connections and communications

Ongoing stakeholder engagement and existing communication platforms among county stakeholders facilitated implementation. Key stakeholder forums included county Health Management Teams (HMTs), which made decisions on HPV vaccination implementation within counties and applied best practices from other counties. Key implementers (eg, county EPI logisticians) mobilised partners to support HPV vaccination initiatives, mobilised clients and followed up with NVIP to obtain vaccination supplies.

We normally hold our stakeholder meetings where we involve all the departments because we realize that alone we can’t do anything…And that is why we are even able to borrow their vehicles when we are held up. (Decision-maker, Kiambu county).

Joint quarterly technical working group meetings were used to brainstorm, disseminate information and strategise on implementation. MOE stakeholders held steering committee meetings, MOH and other stakeholders communicated using WhatsApp groups, while sensitisation activities were conducted by joint MOE-MOH stakeholders. HPV vaccination discussions were included in various stakeholder meetings including parent-teacher associations, ministry of social services and Kenya National Immunization Technical Advisory Group to facilitate integration in school health programmes. The community health strategy facilitated community entry, mobilisation and information dissemination through CHPs, community health assistants, administration officials and religious leaders.

#### Compatibility and relative priority

HPV vaccination leveraged school health programmes and was integrated into routine services, particularly childhood immunisation, health campaigns and partner-supported outreaches. Stakeholders perceived that HPV vaccination fit well within existing services, leveraged available opportunities and resources (eg, staff) and addressed resource shortages, facilitating its scalability and sustainability.

However, there were concerns that HPV vaccination integration required advocacy to generate demand, which was limited within facilities. Stakeholders also reported inadequate prioritisation of HPV vaccination by the MOH during rollout, lower prioritisation within healthcare facilities compared with other vaccinations and limited facility-level planning, which hindered implementation. Furthermore, competing priorities prevented parents from taking girls for vaccination. Additionally, a lack of adolescent-friendly spaces for vaccination delivery within MCH clinics and the additional workload associated with administering HPV vaccination resulted in long waiting times and discomfort among girls.

My daughter…when I took her for the vaccination…we did the normal queue so that I get to hear her feedback…I carried along her cousin, and they told me freely, “Mum why are we sitting next to pregnant people?” And people were just removing their breasts to breastfeed…They were feeling insulted, having sat there for some time…in a government hospital. (National-level decision-maker).

#### Available resources

Information, education and communication (IEC) materials, vaccine supplies, refrigerators, cooler boxes, documentation tools and commodities facilitated delivery within facilities and schools. Some resources were leveraged from the EPI, while others were mobilised through donors and implementing partners. Co-financing from the Global Alliance for Vaccines and Immunization facilitated the procurement of HPV vaccines, while the government covered the operational costs. However, some facilities often lacked vaccine stocks, while others lacked cold-chain infrastructure and did not stock vaccines on-site.

Some facilities…do not stock that [HPV] vaccine…for example…us there was a day someone wanted [HPV vaccine] but we didn’t have [it]. So, we could get these girls and now we were doing referrals…for example sending them to [Facility X] or [Facility Y]. We were not sure if they were going for it. (Nursing officer, Kisumu county).

#### Access to knowledge and information

Initial training for national, county and subcounty stakeholders provided evidence on the need for HPV vaccination, as well as guidelines, standard operating procedures and epidemiology of HPV and cervical cancer. Thereafter, national trainers of trainers cascaded training to HCPs through county and sub-county HMTs. The training contributed to the acceptability of and preparedness for HPV vaccination implementation across counties.

After…the training, they [stakeholders] were ready and accepted to do the roll out. This is after they’d been sensitized, and they were now…able to disseminate the same knowledge to the target population. (Community health services Focal person, Kisumu county).

However, knowledge gaps among key implementers, paired with insufficient and inconsistent training, were reported, especially among HCPs. Some HCPs and community leaders only received basic information (eg, dosage, administration site and eligible age). HCPs also reported a lack of refresher courses and comprehensive continuing medical education, which led them to rely on on-the-job training, brief IEC materials and self-research.

### Individuals’ characteristics

#### Capability and motivation

Most trained stakeholders were supportive of HPV vaccination and were more confident in implementing and advocating for vaccination than untrained stakeholders. Notably, HCPs and CHPs reported a shift in attitudes from hesitancy to advocacy after being sensitised and provided with evidence. Most HCPs were confident and motivated to administer the vaccine and provide education to clients, as they felt it was part of their duties. HCPs, teachers and CHPs reported a strong commitment, often going beyond duty to support HPV vaccination (eg, using personal funds for airtime and transport, conducting follow-ups through WhatsApp messages, home visits and prioritising HPV vaccination for girls attending facilities).

Whenever a mother comes to her regular clinics for…infants below five years, I always ask them, “Do you have any child between 9 to 14 years old?” When they respond, yes, that is when we start talking…I…go to their house to vaccinate the kids if it is difficult for them to come to the hospital. (Nurse, Kisumu county).

Incentives, including transport and lunch allowances (KES 500–1500 (~US$4–US$12)) provided by NGOs, and exempting vaccinating staff from routine duties during outreaches enhanced motivation among CHPs and HCPs. However, hesitancy persisted among some teachers, parents, religious leaders and HCPs due to workload concerns, lack of incentives and limited knowledge and engagement, which reduced willingness to participate in HPV vaccination.

### Implementation process

#### Planning

At the national level, planning for HPV vaccination was conducted by MOH and MOE stakeholders. At the county level, planning was coordinated by the county government through subcounty public health nurses and public health officers who informed facility management teams. These teams then liaised with school management and HCPs to conduct outreaches.

The county government…we are told from this time to this time you’ll have to give out the vaccine…we make…a timetable on the surrounding schools…which days are best to go to which school, we reach out to them so that they can…get consent from the parents…we plan…and…go and give the immunization. (Nurse, Kiambu county).

Challenges to planning school-based vaccination included scheduling conflicts due to untimely communication between schools and facilities, rigid school timetables that disrupted or led to postponement of vaccination outreaches and the 6 monthly vaccination schedule, which hindered client tracking and follow-up.

#### Engaging

Initial stakeholder engagement by the MOH/NVIP was conducted through training targeting key decision-makers at the county, subcounty and national levels. These stakeholders then sensitised the public during subsequent vaccination campaigns. Informational materials were also provided in facilities. Community gatekeepers, including teachers, CHPs and religious leaders, were later sensitised following initial uptake challenges. At the community level, information was disseminated through social media, television and radio. Currently, CHPs conduct community mobilisation and sensitisation through community dialogue forums and events, chiefs’ *barazas* (meetings), schools and churches. HCPs also provide information, encourage mothers to bring girls for vaccination and deliver health education to girls before vaccination.

In schools, we created time to talk to them [girls]…I still think we have a lot of myths and misconceptions…they don’t have the right information…they tell you, “My parents have refused…I don’t know how it would react to the medicine I am taking, I heard that when you are given you can die, you will not deliver [give birth].” (Decision-maker, Kisumu county).

However, inadequate early engagement and training of subnational and community stakeholders, as well as limited community sensitisation before rollout, resulted in low uptake.

#### Doing, reflecting and evaluating

Stakeholders submitted daily reports and monthly summaries for vaccinations conducted during school outreaches and within facilities. The data were used to assess HPV vaccination uptake and plan additional outreaches.

You must report, “We have gone to this school and we have given this number”, so that…she [sub-county public health nurse] can now…plan for the second dose…The data that we get…relates to the vaccines…you order vaccines depending on the numbers that you have administered. (Nursing officer, Nairobi county).

However, HCPs and CHPs perceived that data shared upward were rarely used for local decision-making. Limited monitoring and evaluation structures, lack of performance targets and inadequate feedback mechanisms affected implementation of outreaches. Furthermore, initial school-based vaccination rollout was conducted hurriedly, without adequate community sensitisation and alongside higher-priority initiatives (eg, malaria vaccination in Kisumu county). Consequently, more attention was given to these initiatives, affecting HPV vaccination.

### Characteristics of systems

#### Systems architecture

Currently, Kenya’s health service delivery function is devolved to counties. Therefore, the national government only provided the vaccines, while counties were responsible for sourcing additional HPV vaccination supplies. Implementing partners contributed the largest share of financing, while government co-financing supplemented donor support.

However, stakeholders reported a lack of budget for HPV vaccination within the MOE. Similarly, at county levels, vaccination supplies were often inadequate. County-level policymakers attributed this to difficulties accessing funding for accelerated activities that had not been budgeted for in advance, resulting in delays or postponements of HPV vaccination activities. Limited immunisation budgets across counties and the perception that the national government should provide all vaccination resources led to a heavy reliance on donors.

The belief is that the national is providing everything…that perception makes us at the county to wait for what the national gives, yet health has been devolved…The vaccines are brought by the national…but the other supporting supplies…have to be bought by the county and yet that budget is rarely there. (Decision-maker, Kisumu county).

#### Strategic policy alignment

Goodwill and support from national-level and county-level stakeholders, NGOs, facility and community leaders facilitated HPV vaccination implementation. Key enablers included written memos from the MOE permanent secretary, circulated at county and subcounty levels, to notify schools and request facilitation of access for HPV vaccination. The MOH facilitated HCP training and the employment of CHPs, who supported mobilisation. Across counties, county executive committee members for health and members of county assembly provided transport during outreaches and engaged CHPs to conduct mobilisation and obtain consents from teachers and parents. Subcounty and facility-level leadership supported and coordinated outreaches and health education in collaboration with CHPs. High-level political support was also reported from the former president, MOH and MOE cabinet secretaries, female members of parliament, county governors and first ladies. Notably, counties with strong leadership interest highly prioritised HPV vaccination implementation.

Some counties’ uptake looks better because of the energy they’re putting in. And…continuous education to their health workers at MCH…We have counties with very proactive KEPI team…they will aggressively be able to…do campaigns around the schools…counties that are doing better look like there is more proactiveness in going to school programs. (Decision-maker, Nairobi county).

Key challenges included a limited understanding of the link between HPV vaccination and cervical cancer among community leaders. Furthermore, stakeholders relied on the Kenya Health Information System and national population census data to estimate the number of eligible girls, but these data were often insufficient, hindering planning. Additionally, county-level politicians exerted considerable influence over the healthcare sector; therefore, their goodwill and prioritisation influenced implementation.

## Discussion

Our study explored the drivers of HPV vaccination from the perspectives of multiple stakeholders involved in HPV vaccination in Kenya. Key facilitators included strong global evidence, adaptable delivery strategies, support from programme partners and engagement of community leaders. Major barriers included limited funding for outreaches, late engagement of key stakeholders and community hesitancy driven by myths and misinformation. Parental consent requirements and poor planning disrupted school-based vaccination, while insufficient demand-creation and a lack of adolescent-friendly spaces affected facility-based delivery.

Our findings align with prior studies that identified knowledge and beliefs among key stakeholders as critical facilitators and gaps in training as key barriers to HPV vaccination delivery and uptake.^36,37^ School-based vaccination was perceived as more effective because it facilitated access to the target population. Similar to Kenya, most LMICs have implemented school-based strategies to achieve high coverage because of their convenience and fewer logistical challenges for girls and parents.^38–40^ However, these settings have also reported challenges with school-based delivery, including logistical, infrastructural and coordination issues.^41^ Studies from Uganda, Tanzania and Malawi also highlighted sociocultural beliefs and misinformation, especially concerns about infertility, as major barriers to HPV vaccination.^42,43^ This underscores the need to collaborate with trusted community leaders and influential stakeholders to build trust and enhance community acceptance.^44^

COVID-19 further complicated HPV vaccination by reducing access, diverting resources and increasing hesitancy because of confusion with COVID-19 vaccines. Other studies have reported reduced HPV vaccination uptake and increased vaccine hesitancy during the COVID-19 pandemic.^45,46^ This underscores the need for clear communication strategies and messaging, as well as building health system resilience, to address hesitancy and sustain vaccination programmes during public health emergencies.^44^ Similar to our findings, other studies have also reported limited vaccination infrastructure as a major facility-level barrier.^41,47,48^ Additionally, while stakeholder engagement improved over time, the initial exclusion of key stakeholders limited buy-in. These findings reinforce the need for early engagement and training of frontline HCPs and influential stakeholders, while addressing their needs and concerns, to sustain implementation.^49^

Our findings highlight the need to strengthen multilevel, context-specific and adaptable strategies to improve HPV vaccination in Kenya.^50^ Evidence-based approaches, such as bundled interventions including client education, provider training, assessment and feedback and reminder systems, have shown promise in increasing vaccination uptake.^51^ Leveraging multiple delivery platforms, including healthcare facilities and community settings, while ensuring adolescent-friendly services, could maximise efficiency, enhance access and increase acceptance among girls.^52^ Strong political commitment, sustainable financing and cross-sectoral collaboration could help overcome systemic barriers and scale up HPV vaccination.^53^ Addressing misinformation through clear, targeted communication, for instance through the use of champions, could reduce hesitancy and improve vaccination acceptance.^54^ Additionally, scaling up the WHO-recommended single-dose vaccination schedule, which Kenya adopted in 2025, could reduce follow-up and logistical barriers while improving cost-effectiveness.^55,56^

Our study has strengths and limitations. We obtained insights from multiple stakeholders across different health system levels, providing a comprehensive understanding of the drivers of implementation within the Kenyan healthcare system. The use of the CFIR framework provided a structured, multilevel analysis that captured contextual drivers of HPV vaccination. However, we focused on only three counties, which may not be representative of the entire country. Additionally, some stakeholders were recruited based on other participants’ recommendations, which may have excluded other important stakeholders. Key stakeholders, particularly policymakers and HCPs, may also have portrayed implementation efforts more positively due to social desirability bias.

## Conclusions

Leveraging drivers of successful delivery to inform future programme implementation and addressing barriers through sustained community sensitisation, stakeholder engagement and training and improved resource allocation will be critical for enhancing HPV vaccination coverage. Additionally, adapting context-specific strategies will be essential to achieving and sustaining progress towards cervical cancer elimination in Kenya and similar settings.

## Supplementary material

10.1136/bmjph-2026-005069 online supplemental file 1

10.1136/bmjph-2026-005069 online supplemental file 2

10.1136/bmjph-2026-005069 online supplemental file 3

## Data Availability

Data are available upon reasonable request.
