# Supplementary material for: Multiple-level stakeholders’ perspectives on barriers and facilitators of HPV vaccination delivery in Western and Central Kenya: a qualitative study
Source: BMJ Public Health. 2026 Oct 8;4(4):e005069. doi: 10.1136/bmjph-2026-005069 (PMC13656144; doi:10.1136/bmjph-2026-005069)
Supplement: Supplementary file 1 [file bmjph-4-4-s001.pdf]

DECISION MAKERS, HEALTHCARE PROVIDERS & IMPLEMENTERS IN DEPTH INTERVIEW GUIDE

Instructions for the interviewer:

- Key informants are providers, policymakers in various counties and the national Ministry of Health that are responsible for overseeing implementation of cervical cancer prevention policies and guidelines including HPV vaccination.
- Regarding our study objective, the interviewer should use, and adapt, if necessary, the lines of inquiry below to elicit participants' views on:
    - The delivery of HPV vaccines
    - The overarching health system and policy environment,
    - The implementation context (including barriers and facilitators)
    - Their capacities, and motivations

Name of interviewer: ______________________________
Date: ______________________________
ID of Key Informant:<br>*Eg: NBO/MOH/NIP/KI001 - Nairobi/ Ministry of Health/National Immunization Program/ Key informant #1*<br>______________________________
Job Title of Key Informant: ______________________________
Level of health system where Key Informant is employed (if county, put name of county): ______________________________
Number of Years Key Informant has been in that job: ______________________________
Location of the interview (e.g., office, phone, clinic): ______________________________
Duration of the interview (minutes): ______________________________

Introduction/Opening Statement:

[READ ALOUD – INTERVIEWERS CAN PARAPHRASE]:

Thank you for taking the time to participate in this in-depth interview on the factors that may facilitate or hinder the implementation of HPV vaccine among AGYW in Kenya.

The Kenya Ministry of Health (MoH) recommendations for HPV vaccination is a two-dose schedule 0 and 6 months similar to the World Health Organization Recommendations. Kenya currently has a national HPV vaccine program based on evidence with school-based vaccination for 9-10 years which was initiated in 2019. GAVI has supported two HPV vaccination demonstration projects from 2013-2015 and 2016-present in Kitui county (outside of our proposed study areas). Both

demonstration projects used the quadrivalent HPV 16/18/6/11 vaccine in a school-based strategy that targeted girls aged 9-10 years, and the first achieved 85% coverage.
We aim to understand the key barriers and facilitators currently faced in the roll out of the HPV vaccination program. We are conducting this study to generate insights which will inform the MOH process for implementation. We are interested in your perceptions of HPV vaccine acceptability, the factors that you think may help or hinder health care workers and teams tasked with implementing HPV vaccine provision effectively and make it a sustainable component of cervical cancer prevention among AGYW in our communities.

This interview will take approximately 1h 30min. With your permission, I would like to record the interview on a digital recorder [show the participant the digital recorder]. The recording will be kept securely and confidentially, and only used for the research purposes as described in the informed consent form [show the participant the form, which s/he has already signed]. Do I have your permission to record this interview?

INTERVIEWER SHOULD CONFIRM WITH THE KEY INFORMANT THAT S/HE HAS PERMISSION TO RECORD THE IDI ON A RECORDED DEVICE. IF RESPONDENT CONSENTS, THE INTERVIEWER CAN START RECORDING NOW. RECORD THE TIME AT WHICH THE INTERVIEW BEGINS.

START TIME OF IDI: ______________________

Interview:

0. Introduction question
Could you describe your involvement with HPV vaccination?
<u>*Probe:*</u>
- Where do you work, what is your position?
- What is your role in this space (either formal job or community leadership role)?
- The duration of your work in this space?

1. Intervention Characteristics
<u>1.a. Intervention Source [*for program implementers/decision makers only*]</u>

i) In your opinion, how did external organizations/players influence the development of the Kenyan national HPV vaccination program (i.e., where did the program originate from)?

[External Policy & Incentives]
ii)What international policies/ mandates have influenced the decision to roll out the HPV vaccine in Kenya/ your setting?
ii) How did external financial or other incentives influence the decision to implement the HPV vaccination in your setting?

<u>*Probe:*</u>
→ *Was the reduced cost of vaccines through GAVI, influence the decision? How about the need to improve performance on cervical cancer elimination goals (90-70-90)?*

1.b. Evidence strength and quality

i) What evidence were you provided with supporting the introduction of the HPV vaccine in Kenya? How sufficient was this evidence?
ii) What do you and other influential stakeholders in Kenya think of the HPV vaccine?
iii)What other evidence is needed on the HPV vaccine to get stakeholders to support the national scale up?

Probe:
- → *Quality concerns?*
- → *Validity of evidence?*

1.c. Adaptability

i)In your opinion, how can the HPV vaccine delivery be adapted to the different settings in Kenya? What adjustments can be made to meet local needs?

[Note to interviewer*: Some adaptation may include leveraging schools, vaccination campaigns and other strategies to reach girls who are in schools and those out of school.]*

ii) What changes are needed for the HPV vaccine to be effectively rolled out in your setting/(health system for the MOH/ MOE) (e.g., infrastructure, training, staffing etc.)
iii) How feasible are the proposed adaptations?
iii) Who decides the changes needed for effective roll-out of HPV vaccination in your setting/ Kenya? How are these decisions made?
iv) **[Planning]**
In your opinion, how was the planning process for the implementation of the HPV vaccine program carried out at your facility, district, county, nationally?
v) In your opinion, what needs to be done to improve uptake of HPV vaccine?

1.d. Trialability [*for program implementers/decision makers only*]
[*Note to interviewer:* *Please confirm if the interviewee is familiar with the Kitui demonstration project. If not, read: GAVI has supported two HPV vaccination demonstration projects from 2013-2015 and 2016-present in Kitui county. Both demonstration projects used the quadrivalent HPV 16/18/6/11 vaccine in a school-based strategy that targeted girls aged 9-10 years, and the first achieved 85% coverage.***]**

i)[If yes,] What do you know about the demonstration project that was implemented in Kitui?
ii) [If no,] What do you think about the Kitui demonstration project?
iii)In your opinion, how helpful do you think was the Kitui demonstration project to the national vaccine rollout? If yes how? If not, why not?

Probe:
- ➔ *The ability to test HPV vaccination roll out at small scale*
- ➔ *The ability to reverse course if warranted*

1.e. Complexity

i) In your opinion, how complicated is the delivery of the HPV vaccine in your setting/ Kenya?

ii) How complex was the implementation of the HPV vaccine delivery during the demonstration projects? (*i.e., in terms of resource allocation, uptake, roll out and integration in the health system, scale up and sustainability, number of steps required to implement HPV vaccination)?*

1.f. Design Quality & Packaging

(i) How are HPV vaccination data/outcomes presented to policymakers here in Kenya?

ii) How do these affect implementation of HPV vaccination?

iii) **[Available Resources]**

What informational/ educational/ marketing materials are available on HPV vaccination in your setting/ Kenya? How do these materials affect the national roll out?

iv)How is public health education on the HPV vaccine undertaken in your setting/ Kenya?

Probe:

- ➔ *Was the public educated on the vaccine before its introduction into health facilities?*
- ➔ *How long was the public educated on the vaccine before its introduction into health facilities?*
- ➔ *How was training done for HCWs on the importance of HPV vaccine implementation prior to the introduction of HPV vaccine in health facilities?*
- ➔ *How were community gatekeepers approached prior to the introduction of the HPV vaccine in health facilities?*
- ➔ *How is the continued community engagement post introduction of the vaccine being implemented?*

1.g. Cost

i) What is the cost of providing full HPV vaccination to girls in Kenya/ your setting?

Probe: Considering all possible costs involved e.g., service, staff, travel costs etc.

ii) How much does it cost to implement HPV vaccination in government health facilities/ your area?

iii) Which resources do you use/ need to deliver HPV vaccines to AGYW in your setting/ county? (*e.g., clinic rooms, staff, storage facilities, cold chain, etc.*)

iv) How are these resources mobilized?

v) What gaps in resources, if any, have you experienced in your setting?

vi) How have these gaps been addressed in your setting?

vii)Which other solutions would you suggest to address these gaps?

Probe:

- ➔ *Percentage of the facility/ county/ national health budget allocated to HPV vaccine roll-out (e.g., transport and storage of HPV vaccines, staff allocations, community education in television, radio and social media, staff and personnel training)*

2. Outer Setting

2. a. AGYWs Needs & Resources

i) What barriers do AGYW face to receiving the HPV vaccine?

ii)Which positive and negative experiences have you heard from AGYW about the HPV vaccination program?

iii) In your opinion, to what extent are implementers aware of the needs and preferences of AGYWs?

iv) In your opinion, to what extent have AGYWs' needs been taken into consideration during the implementation of the HPV vaccination program in your setting/ Kenya?

2.b. Cosmopolitanism [*for program implementers/decision makers only*]

i)How has the diversity of different counties impacted the effective rollout of the HPV vaccination program for AGYW?

ii)How does networking and information exchange take place between the Kenyan MOH and other Ministries/ county managers during the HPV vaccine roll out?

iii) How do you exchange information with other stakeholders outside your setting?

2.c. Peer Pressure [*for program implementers/decision makers only*]

i)How have other countries/ counties in the region implemented HPV vaccination national rollouts?

ii)How has this impacted Kenya's/ your setting's implementation of the HPV vaccination program?

3. Implementation context (Inner Setting)

3.a. Structural Characteristics

i)How does the existing infrastructure (maturity, size and coverage) of the MOH/ the county/ your facility affect the delivery of the HPV vaccine for AGYW?

iii)How does the current organization of health services in your setting support or hinder the implementation of the HPV vaccination program for AGYW?

3.b. Network & Communications

i)Who are the influencing/ main stakeholders in HPV delivery in your setting?

ii)How does information flow between them?

iii) Can you describe your working relationship with the influential stakeholders?

Probe:

→ *What is the composition of the network and how does the information flow within this network at county or national level depending on your level of involvement in the HPV vaccine delivery*

3.c. Implementation climate

i)Do you think there was interest or hesitation to implement HPV vaccination at the national level or your setting?

ii) How did the health managers and healthcare workers in Kenya/ your setting receive the delivery/ rollout of the vaccine?

iii) **[Compatibility sub-construct]**
Do you think the HPV vaccination program for AGYW fits well with existing work processes and practices in Kenya/your setting? Please explain?

iv) **[Relative Priority]**
To what extent might the implementation of the HPV vaccination program for AGYW take a backseat to other high-priority initiatives going on now in Kenya or your setting?

v) **[Learning sub-construct]**
To what extent are you/ your setting able to try new things to improve HPV vaccination delivery processes for AGYW?
vi) Does the HPV vaccination align with community priorities?

3.d. Readiness for implementation
i) **[Leadership Engagement sub-construct]**
In your opinion, what has been the level of support from leaders in your setting for the delivery of the HPV vaccine for AGYW?
ii)What are some of the things that leaders in your setting have done to support HPV vaccine delivery?

iii) **[Available resources]**
What kind of resources, support or actions do you need to make HPV vaccine delivery successful in your setting?

Probe:
- ➔ *Are you ready to provide this support? If yes, why? If no, why not?*
- ➔ *Which other needs does your facilities/ community have (training, materials, information, community awareness)? Are you prepared to make them available? Why?*

4. Process
4.a. Planning [*for program implementers/decision makers only*]
i)Does your setting have an implementation plan for HPV vaccine delivery?

***[Note to interviewer**: Aspects of the implementation plan may include health-facility based delivery, targeting girls aged 9-14 years, etc.]*
ii)What is the HPV vaccination implementation plan/ strategy in your setting/ Kenya?
ii) What role has the plan for implementation played during implementation?

4.b. Executing [*for program implementers/decision makers only*]
ii) To what extent has the HPV vaccination program been implemented according to the implementation plan in your setting?

4.c. Engaging
[Opinion Leaders/ Champions]

i)Who are the key people who have been engaged in the HPV vaccine delivery in your setting/ nationally?
ii) Other than healthcare providers and employees of MOH, who are other advocates/champions of HPV vaccination in your setting/ nationally? (E.g., teacher, community leaders, etc.)
iii)How do people perceive these champions?

[Formally appointed internal implementation leaders]
i)Who leads the implementation of the HPV vaccine delivery in your setting? (E.g., nationally, at county or community level)
ii)Who else is involved with leading the implementation?

4.d. Reflecting and Evaluating [*for program implementers/decision makers only*]

i)How has the rollout of HPV vaccination been monitored and evaluated nationally/ in your setting?
ii)How has the feedback/ data from the monitoring been used to inform the national/ local strategy?
iii)How is progress towards implementation goals being assessed nationally/ in your setting?

5. The Health system and policy context

5.a. Health systems context

i)How do you think the rollout of the HPV vaccination is going on in your setting/ community/the health system?
ii) What knowledge and beliefs about the HPV vaccine impacted implementation? (*At facility, district, county, national, levels*)?
ii) Reflecting on the factors in your setting (health system for MOH), is there anything else that is affecting HPV vaccine implementation positively or negatively?

Probe:

- ➔ *Supply chain?*
- ➔ *Human resource shortages?*
- ➔ *Financial resources?*
- ➔ *Vaccine hesitancy*
- ➔ *Awareness of the cervical cancer prevention measures?*

5.b. Policies and guidelines

i) Are you aware of any policies, programs, or guidelines that might affect the delivery and uptake of HPV vaccines in your setting?

Probe:

- ➔ *What are they?*
- ➔ *How might they affect implementation? Scalability? Sustainability?*
- ➔ *How about government/ community directives?*

Thank you for participating in this interview. I have no further questions. Before we end the interview, is there anything you'd like to add?

[If yes, listen and continue recording, if no, stop recording and end the interview].

END TIME OF IDI: _______________________
