# Supplementary material for: Multiple-level stakeholders’ perspectives on barriers and facilitators of HPV vaccination delivery in Western and Central Kenya: a qualitative study
Source: BMJ Public Health. 2026 Oct 8;4(4):e005069. doi: 10.1136/bmjph-2026-005069 (PMC13656144; doi:10.1136/bmjph-2026-005069)
Supplement: Supplementary file 2 [file bmjph-4-4-s002.pdf]

## DECISION MAKERS, HEALTHCARE PROVIDERS & IMPLEMENTERS CODEBOOK

| CFIR Domain | Code Name | Code Description | Code Example |
|---|---|---|---|
| **A. Individuals**<br><br>Apply this code to any intervention characteristic factors that do not fit with other sub-codes in the "A" group. | A1. Roles | Apply this code to any description of participants' role in HPV vaccination, including where they work, their position in either formal job or community leadership, and duration of work in their role. | *"I am the MCH (Maternal Child Health) in-charge, I oversee the services in this facility in this MCH department, and also, I participate in the clinical work like now the vaccination, monitoring of the vaccines including quantity of the vaccines."* (KBU-HCP-KI004) |
| | A2. High-level Leaders | Any mention of recognized stakeholders who lead HPV vaccination delivery in participants' settings, including leaders at national, county, and community levels. | *"The public health officers organize meetings, and they are the ones who organize activities. When we are on the ground, if you have any problem about insecurity, we inform the chief." (KBU-OPL-KI001)* |
| | A3. Opinion Leaders/Champions | Any mention of the key people engaged in HPV vaccination in Kenya/participants' settings, other advocates of HPV vaccination other than healthcare providers and employees of MOH, and how people perceive these champions. Include any suggested champions, with reasons. | *"In the community apart from the CHWs [Community Health Workers], maybe the gatekeepers. The gatekeepers and even the youth leaders and even the admin…Gatekeepers are the community watch dogs…" (KBU-OPL-KI007)* |
| **B. Intervention characteristics**<br><br>Apply this code to any intervention characteristic factors that do not fit with other sub-codes in the "B" group. | B1. Innovation Source | Any mention of groups or organizations that developed or sponsored the implementation and rollout of HPV vaccination programs in Kenya/participants' settings. Include any mention of international policies and mandates, cervical cancer prevention targets, external financial or other incentives, decision-making processes, and specific bodies/organizations that were involved in HPV vaccination programs. | *"So, when I was joining my facility, I met the vaccination ongoing but along the way especially when we were going for the outreaches, we were usually supported by the NGOs like CHAI (Clinton Health Access Initiative)." (NBO-HCP-KI004)* |
| | B2. Innovation evidence base | Any mention of evidence presented to participants supporting introduction of HPV vaccination in Kenya, whether the evidence was sufficient, what influential stakeholders think about effectiveness of the HPV vaccine, and other evidence needed by stakeholders to support national scale-up. Include any mention of trainings and information provided/not provided to | "*We don't have that experience already in Kenya. Maybe soon or later when this now happens when these girls now grow to age, and you find that research is done amongst them, and you find maybe none of them or very few are getting the cervical cancer, then it would be evidence enough for the next generations." (KSM-DCM-KI003)* |

| | | | |
|---|---|---|---|
| | | stakeholders prior to involvement in vaccination programs. | |
| | B3. Innovation adaptability | Any mention of how HPV vaccine delivery can be modified, tailored or adapted to fit different settings in Kenya, including suggestions on changes needed to meet local needs, whether the changes are feasible, how HPV vaccine delivery has been done in participants' settings, changes needed for effective HPV vaccination rollout, who makes decisions on changes, and how decisions are made for effective HPV vaccination in participants' settings. | "*We can do outreaches in school because most of the population that we target are found in school. The hours we want to vaccinate them is also again, they're in school during those hours. And then we also have a population that is out of school, and they're out there in the community. So, we also target them in terms of outreach in the community.*" (NBO-DCM-KI004) |
| | B4. Innovation trialability | Any mention of participants' knowledge and opinion on the Kitui demonstration project, and whether the project was helpful to the national vaccination rollout, including reasons. | "*The demonstration itself was successful. It was successful in terms of acceptance, in terms of uptake, and all that. So, it helped a lot. And we were guided by the findings from that demonstration into now planning for the implementation.*" (NBO-DCM-KI004) |
| | B5. Innovation complexity | Any mention of whether HPV vaccination delivery is complicated in the participants' settings/nationally, including the complexity of HPV vaccine delivery during the demonstration projects, with reasons. | "*It is not complicated yes if you go through the right channel and create awareness. It is not complicated like if you create awareness and everyone gets to know about it, we can do it.*" (KSM-HCP-KI004) |
| | B6. Innovation design | Any description of how HPV vaccination is designed and packaged, including how data/outcomes are collected and presented to policymakers in Kenya/participants' settings, and how the outcomes affect HPV vaccination implementation, with reasons. Include any suggestions on how data/outcome should be presented. | *"The data is captured through KHIS (Kenya Health Information System), you know, that, however, KHIS does not capture all the data maybe which would really help you like what you're asking."* (NBO-DCM-K1003) |
| | B6.1 Education and information material | Any mention of available informational and educational material on HPV vaccination in Kenya/participants' settings, whether the material affect national HPV vaccination rollout, and how public education on HPV vaccination is/was done in Kenya/participants' settings. | "*Like in our facility there are occasions when we do the health talks, like in our facility we do it twice a week, Tuesday, and Thursday morning yes. So, there are those health talks and the if the government would use the media because in Kenya, we are people who are visual mostly but reading* |

| | | | |
|---|---|---|---|
| | | Include any suggested information/public education material/activities. | *materials we are not very good at that."* (KSM-HCP-KI006) |
| | B7. Innovation cost | Any mention of cost of providing full HPV vaccination to AGYW in Kenya/participants' settings, cost of implementing HPV vaccination in health facilities, resources used or needed to deliver HPV vaccination to AGYW in participants' settings, how vaccination resources are mobilized, experienced gaps in resources, how gaps are addressed, and suggested solutions to address resource gaps. | *"I mean funds should be mobilized because a lot of these things will need funds and if the government doesn't have funds the community doesn't have funds too that's why I will emphasize that we need like-minded organizations to come and help to roll out this thing, the way they did it with the other vaccines and especially let me use malaria is one of them."* (KSM-OPL-KI003) |
| **C. Outer Setting**<br><br>Apply this code to any outer setting factors that do not fit with other sub-codes in the "C" group. | C1. Local attitudes | Any mention of barriers AGYW face to receiving HPV vaccination, positive or negative experiences from AGYW about HPV vaccination, whether vaccination implementers are aware of needs and preferences of AGYW, and whether needs of AGYW have been considered during HPV vaccination implementation in Kenya/participants' settings. | *"Yes, when we get a child has a consent, but she is personally saying she doesn't want, you don't force, we just note in that sheet, the consent form, 'not given' 'declined', so she will deal with the parent. If the parent can bring her is okay, and those who are sick also we don't give." (KSM-HCP-KI007)* |
| | C2. Partnerships & Connections | Any mention of how the diversity of counties impacted HPV vaccination rollout for AGYW, networking and information exchange between MOH and other ministries/stakeholders during HPV vaccination rollout, and how participants exchange information with other stakeholders outside their settings. Can be double-coded with C3. | *"Yeah, the diversity really affects because like in Kenya if you go to, if you go to the northeastern, they don't take vaccines completely." (KSM-DCM-KI003)* |
| | C3. Policies & Laws | Any mention of policies, programs, or guidelines that might affect the delivery and uptake of HPV vaccination in participants' settings, including how they might affect implementation, scalability, and sustainability of HPV vaccination programs. Also include any suggestions on required policies or guidelines. | *"What I want to be in the policy is what I have shared… Like that one of digitizing and putting it is NEMIS so that automatically we are able to get all the girls who are eligible for the vaccination." (KSM-DCM-KI003)* |
| | C4. External Pressure | Any mention of how other countries/counties in the region implemented HPV vaccination rollouts, and whether other countries/counties' | *"I think the biggest difference, we are seeing this in terms of strategies. One, there are counties that also have… there are counties, I can't remember which one of* |

| | | | |
|---|---|---|---|
| | | implementation influenced HPV vaccination programs in Kenya/participants' settings. Can be double-coded with C2. | *them has opened facilities over the weekend for the girls. That means they have been very sensitive to the need of the girl." (NBO-DCM-K1002)* |
| **D. Inner Setting**<br><br>Apply this code to any implementation context (inner setting) factors that do not fit with other sub-codes in the "D" group. | D1. Structural Characteristics | Any mention of how existing infrastructure of the MOH/county/facility/participants' setting has affected the delivery of HPV vaccination for AGYW, and how current organization of health services in participants' settings support or hinder the implementation of the HPV vaccination program for AGYW, including reasons. | *"Let's talk about our facility, you find that where the vaccine is being administered and where we have the gynecology clinic, it is in the same setup. So, you find that when these girls come for the vaccine, they find that there is a long queue for the cervical cancer screening, so they just get worried about the same." (NBO-HCP-KI006)* |
| | D2. Relational Connections & Communications | Any mention of the key influencing/main stakeholders involved in HPV delivery in participants' settings, composition of stakeholder networks at county/national/participants' settings, how information flows between influential stakeholders, and participants' working relationships with the stakeholders. | *"The first one I think is parents' coz those are the major stakeholders; they are the ones who are very close to the girls than the medical officers and religious leaders." (KSM-OPL-KI008)* |
| | D3. Culture | Any mention of whether there was interest or hesitation to implement HPV vaccination in Kenya/participants' settings, how healthcare managers/providers/participants' settings received the HPV vaccination delivery/rollout, including reasons. | *"Hesitation was there just like you said earlier when the Catholic bishops came out giving out their ideas, so hesitation was there coz, we also have followers who are Catholics so it was there. Even in our Muslim community it was there coz it was a new thing they had not known it, yes sensitization was done but there were some fears," (KSM- OPL -KI008)* |
| | D4. Compatibility | Any mention of whether the HPV vaccination program for AGYW fits well with existing work processes in Kenya/participants' settings. Include descriptions of where/how HPV vaccination is currently fitted/integrated in the setting, and suggestions for integration, including reasons. | *"Currently like the way we are doing it right now; we have fitted it in CWC [Child welfare Clinic]. So, for those girls who come for the vaccine, they'll always come to CWC, we give it, we actually have integrated it there." (KBU-HCP-KI003)* |
| | D5. Relative Priority | Any mention of whether implementation of HPV vaccination programs for AGYW has taken a backseat to other ongoing high-priority initiatives in | *"We haven't pushed it to the back, but it is at the back because the government has not prioritized it. Look at for instance the Covid vaccine, you saw how much effort* |

| | | | |
|---|---|---|---|
| | | Kenya/participants' settings, including reasons. | *the government put in it to make sure this vaccine is taken, I wish they would have put that much effort on the HPV vaccine too." (KSM-HCP-KI006)* |
| | D6. Available resources | Any mention of the kind of resources, support, or actions participants need to make HPV vaccination delivery successful in their settings, whether participants are ready to provide these supports, other needs facilities/communities/settings have, and whether participants are ready to make the needed support available. | *"Support is just to have someone do the health talks and mobilization at the community and then we want all the resources to be available." (KSM-HCP-KI007)* |
| **E. Implementation Process**<br><br>Apply this code to any 'process' factors that do not fit with other sub-codes in the "E" group. | E1. Planning | Any mention of the implementation plan for HPV vaccination delivery in Kenya/participants' settings, including a description of the HPV vaccination implementation plan/strategy, how the planning process for implementation of the HPV vaccination program is/was done, and the role the implementation plan played during implementation. Include any mention of suggestions on how planning should be done to improve HPV vaccination uptake. | *"We are just starting it as part of our routine. As I've said, we are riding on any other supported activity at the community level. We carry our vaccines there and we also carry out the outreaches at the facility level. Yeah, and we are encouraging those who are sitting at the triage desk to be asking those questions, in fact, we have integrated COVID, and HPV to ask whether this person has received the jab or not." (NBO-DCM-K1001)* |
| | E2. Doing | Any mention of the extent to which the HPV vaccination program has been implemented according to the implementation plan in Kenya/participants' settings described in the E1 code, challenges affecting implementation of HPV vaccination in Kenya/participants' settings, and thoughts on what should be done to improve HPV vaccination uptake. | *"I think to a greater extent, because you go to every health facility in this country, you can get the HPV vaccine. That was the idea. We knew if we go by facility alone, we will not get enough numbers. So, we stock the vaccine in every facility, so that at least that one we have done. Also, the Counties are looking for other alternatives and it's also part of the plan. When we say it can be supplemented by any other strategy. So, I think to a great extent we have." (NBO-DCM-K1002)* |
| | E3. Engaging | Any mention of how key stakeholders became engaged with the innovation, their role in implementation, and whether the engagement strategies were successful. Exclude or double code | "Even at the county level, we've had meetings with education officers, the director, and the regional directors, just to try and make them understand why we need to visit schools to vaccinate |

| | | | |
|---|---|---|---|
| | | with statements in 'A', 'B', and 'D' codes on opinion leaders/champions, trainings and information provided, and general networking, communication, and relationships. | girls, in Nairobi, it is from the age of 10 to 14 years." (NBO-DCM-K1001) |
| | E4. Reflecting & evaluating | Any mention of how the HPV vaccination rollout has been monitored and evaluated in Kenya/participants' settings, how the monitoring feedback/data is used to inform national/local strategies, and how progress towards implementation goals have been assessed in Kenya/participants' settings. | *"I don't think we have ever evaluated-we only evaluated once through that one sitting we had. We have never evaluated again." (NBO-HCP-KI005)* |
| **F. Characteristics of systems**<br><br>Apply this code to any health system and policy context factors that do not fit with other sub-codes in the "F" group. | F1. Systems architecture | Any description of how the vaccination rollout is going on in the community/health system/participants' settings, knowledge and beliefs about the HPV vaccine that affect HPV vaccination implementation, and any other factors in participants' settings that affect HPV vaccination positively or negatively. | *"At least they are trying because at least they are going for the school campaigns once in a while, so they are trying." (KSM-OPL-KI006)* |
| | F2. Strategic policy alignment | Any mention of the support received from leaders by participants/participants' settings for the delivery of HPV vaccination for AGYW, including specific things done by leaders to support HPV vaccination delivery. Also include any mention of lack of support from leadership. | *"Yes, the sub-county team as well has also given us support, organizing the school health programs to the subcounty team, they have done a good job. Like… "Tomorrow we are doing this school…" So, they are the ones to communicate from the sub-county." (NBO-HCP-KI002)* |
| | **G. Barriers and facilitators** | **Apply this code to any mention of barriers and facilitators affecting HPV vaccination implementation in Kenya/participants' settings. May be double coded with "A"- "F" codes.** | |
| | **H. Recommendations** | **Apply this code to any mention of suggestions and recommendations to improve HPV vaccination delivery and uptake in Kenya/participants' settings. May be double coded with "A"- "F" codes.** | |
| | **I. Exemplary quotes** | **Apply this code to any outstanding quotes that occur throughout the transcripts to be used in the final analysis.** | |
